# Plasma Metabolomics of Intermediate and Neovascular Age-Related Macular Degeneration Patients

**DOI:** 10.3390/cells10113141

**Published:** 2021-11-12

**Authors:** Sabrina L. Mitchell, Chunyu Ma, William K. Scott, Anita Agarwal, Margaret A. Pericak-Vance, Jonathan L. Haines, Dean P. Jones, Karan Uppal, Milam A. Brantley

**Affiliations:** 1 Medical Center, Vanderbilt Eye Institute, Vanderbilt University, Nashville, TN 37232, USA; sabrina.l.mitchell@vumc.org (S.L.M.); anita.agarwal@vanderbilt.edu (A.A.); 2 Department of Medicine, School of Medicine, Emory University, Atlanta, GA 30322, USA; machunyu4402@hotmail.com (C.M.); dpjones@emory.edu (D.P.J.); kuppal2@emory.edu (K.U.); 3 John P. Hussman Institute for Human Genomics, School of Medicine, University of Miami Miller, Miami, FL 33136, USA; w.scott@med.miami.edu (W.K.S.); mpericak@med.miami.edu (M.A.P.-V.); 4 Department of Population and Quantitative Health Sciences, Institute for Computational Biology, Case Western Reserve University, Cleveland, OH 44106, USA; jonathan.haines@case.edu

**Keywords:** age-related macular degeneration, IAMD, NVAMD, metabolomics, acylcarnitines, carnitine shuttle, phospholipids

## Abstract

To characterize metabolites and metabolic pathways altered in intermediate and neovascular age-related macular degeneration (IAMD and NVAMD), high resolution untargeted metabolomics was performed via liquid chromatography-mass spectrometry on plasma samples obtained from 91 IAMD patients, 100 NVAMD patients, and 195 controls. Plasma metabolite levels were compared between: AMD patients and controls, IAMD patients and controls, and NVAMD and IAMD patients. Partial least-squares discriminant analysis and linear regression were used to identify discriminatory metabolites. Pathway analysis was performed to determine metabolic pathways altered in AMD. Among the comparisons, we identified 435 unique discriminatory metabolic features. Using computational methods and tandem mass spectrometry, we identified 11 metabolic features whose molecular identities had been previously verified and confirmed the molecular identities of three additional discriminatory features. Included among the discriminatory metabolites were acylcarnitines, phospholipids, amino acids, and steroid metabolites. Pathway analysis revealed that lipid, amino acid, and vitamin metabolism pathways were altered in NVAMD, IAMD, or AMD in general, including the *carnitine shuttle* pathway which was significantly altered in all comparisons. Finally, few discriminatory features were identified between IAMD patients and controls, suggesting that plasma metabolic profiles of IAMD patients are more similar to controls than to NVAMD patients.

## 1. Introduction

Age-related macular degeneration (AMD) is a significant health burden in the aging population. It is the leading cause of vision loss in individuals over age 60, affecting an estimated 150 million people worldwide, and this number is expected to reach 288 million by the year 2040 [1]. The stages of AMD are broadly categorized as early, intermediate, and advanced. Early AMD is defined by the presence of small- or medium-sized drusen deposits without retinal pigmentary changes, while intermediate AMD (IAMD) is marked by larger, more extensive drusen and/or pigmentary abnormalities [2]. Vision loss often occurs in advanced AMD which includes two types: geographic atrophy, in which retinal cells gradually degenerate, and neovascular AMD (NVAMD), characterized by the formation and proliferation of new choroidal blood vessels. The aberrant new vessels in NVAMD cause exudation and macular edema, resulting in retinal damage and severe vision loss. The majority of AMD-related vision loss is caused by NVAMD [3,4]. 

The complex etiology of AMD includes multiple genetic, environmental, and lifestyle factors. Genetic variants at more than 30 loci have been independently associated with AMD risk [5]. Advanced age, smoking, and higher body mass index (BMI) are also established risk factors for AMD [6,7]. Despite the known risk factors, it is difficult to predict which individual patients will progress to advanced stages of this disease.

Metabolomics is the study of small molecules, or metabolites, in biological samples such as cells, tissues, or biofluids. Typically, mass spectrometry- or nuclear magnetic resonance spectroscopy-based approaches are used for either targeted or untargeted studies of the metabolome. Investigating how different health states affect metabolic profiles can provide fundamental insights into disease pathophysiology. Over the last 20 years, metabolomics has been employed in the study of numerous diseases and in some instances has ultimately led to advances in biomedical research [8]. Metabolomics has been increasingly applied to ophthalmologic conditions to identify metabolites or metabolic pathways altered in these diseases [9] including in AMD [10,11,12,13,14,15,16,17,18,19,20,21]. We have previously performed two untargeted metabolomics studies comparing NVAMD patients to non-AMD controls [10,14]. In our pilot study, we observed alterations in amino acids and in features with database matches to glycine-conjugated bile acids and vitamin-D related metabolites [10]. Our second study, performed in a larger, independent cohort, revealed alterations in long-chain acylcarnitines and in features with matches to lysophospholipids and taurine-conjugated bile acids [14].

The goal of the present study was to identify metabolites or metabolic pathways altered in AMD. This study included IAMD and NVAMD patients as well as non-AMD controls, allowing for comparisons at important clinical transitions. High-resolution, untargeted metabolomics was performed via liquid chromatography-mass spectrometry (LC–MS). To identify metabolic alterations in AMD, we compared all patients with AMD (IAMD plus NVAMD) to non-AMD controls. In addition, to determine metabolites and metabolic pathways altered at different stages of disease, we compared IAMD patients to non-AMD controls and NVAMD patients to IAMD patients.

## 2. Materials and Methods

### 2.1. Study Participants

Study participants were recruited from the Vanderbilt Eye Institute between 2002 and 2011. The Vanderbilt University Medical Center Institutional Review Board approved the research study protocols. Research was conducted in accordance with Health Insurance Portability and Accountability Act regulations and adhered to the tenets of the Declaration of Helsinki. Written, informed consent was obtained from all participants prior to study enrollment.

All study participants were of European ancestry and at least 55 years old at the time of enrollment. A retina specialist administered comprehensive eye exams that included slit-lamp biomicroscopy and dilated fundus examination with indirect ophthalmoscopy for all participants. High-resolution color fundus photographs obtained using a Zeiss 450+ fundus camera (Carl Zeiss Meditec, Dublin, CA, USA) were graded using the Clinical Age-Related Maculopathy Scale (CARMS) [22]. The CARMS includes five distinct categories from Grade 1 (no AMD), defined as fewer than 10 small drusen and no macular pigment changes, to Grade 5 (NVAMD), defined as extensive AMD characterized by choroidal neovascularization, subretinal hemorrhage or fibrosis, or photocoagulation scarring consistent with AMD treatment [22]. Grade 3 (IAMD) is characterized by extensive intermediate drusen or any large soft drusen. For all study participants, each eye was assigned an AMD grade from 1 to 5.

In this study, IAMD cases were defined as those patients with Grade 3 in both eyes, while NVAMD cases were defined as those patients with Grade 5 in at least one eye. Controls were those individuals that were assigned Grade 1 in both eyes. The study population included 91 IAMD patients, 100 NVAMD patients, and 195 non-AMD controls. Of the 100 NVAMD patients, 92 had Grade 5 in both eyes, and 8 had Grade 4 (geographic atrophy) in the fellow eye. Among the participants in this study, 100 NVAMD patients and 192 controls were included in our previous metabolomics study comparing NVAMD patients to non-AMD controls [14].

Blood was drawn from each participant at the time of study enrollment. Acetate–citrate–dextrose tubes containing 1.5 mL of Solution A: trisodium citrate 22.0 g/L, citric acid 8.0 g/L and dextrose 24.5 g/L (Becton Dickinson, Franklin Lakes, NJ, USA) were used for sample collection. These tubes were centrifuged for 10 minutes at 4 °C. Plasma was transferred to 1.5 mL conical tubes and immediately stored at −80 °C.

A self-administered questionnaire was used to collect detailed histories of comorbid medical conditions and environmental exposures including height and weight, and diabetes, hypertension, hyperlipidemia, and smoking status. The reported height and weight were used to calculate BMI. Participants who indicated they had ever smoked at least 100 cigarettes were considered smokers, while those who had smoked fewer than 100 cigarettes were considered nonsmokers. Descriptive statistics for demographic, environmental, and clinical variables were calculated for each group in the study population. Welch’s two-tailed t-tests were used to compare continuous variables, and chi-squared tests were used to test for differences in categorical variables.

### 2.2. High-Resolution Untargeted Metabolomics

Plasma samples were thawed and analyzed by LC–MS at Emory University as previously described [10,14,23,24,25,26,27]. Samples were randomized into 20-sample batches that included both AMD patients and non-AMD controls. Plasma aliquots (65 µL) were treated with 130 µL acetonitrile (2:1 *v*/*v*) containing 3.5 µL of an internal isotopic standard mix [10,14,25,26] and incubated on ice for 30 min. Samples were then centrifuged for 10 min (16,100× *g* at 4 °C) to remove protein. The supernatants (10 µL) were loaded onto an Accela Open Autosampler maintained at 4 °C. Each sample was analyzed in triplicate using a Thermo LTQ Velos Orbitrap high-resolution (60,000 mass resolution) mass spectrometer (Thermo Fisher Scientific, San Diego, CA, USA) and C18 column chromatography [14,23,28]. Elution was obtained with a formic acid/acetonitrile gradient at a flow rate of 0.35 mL/min for the initial 6 minutes and 0.5 mL/min for the remaining 4 min. The first 2-minute period consisted of 5% solution A [2% (*v*/*v*) formic acid in water], 60% water, and 35% acetonitrile. The final 4-minute period was maintained at 5% solution A and 95% acetonitrile. The mass spectrometer was set to collect mass-to-charge ratio (*m/z*) from 85 to 1985 over 10 min. Electrospray ionization was used in positive mode for detection. For quality control and assurance, pooled reference plasma was run both prior to and after each batch of samples.

### 2.3. Data Processing and Analysis

Mass spectral data were extracted and processed for all samples following the same protocol. An adaptive processing software package, apLCMS (v6.3.3) (http://web1.sph.emory.edu/apLCMS/), designed for use with high-resolution mass spectrometry data, was used for noise removal and for feature extraction, alignment, and quantification [29]. Each extracted feature is defined by a unique combination of *m/z* and retention time (RT). To enhance the feature detection process and perform quality evaluation, systematic data re-extraction and statistical filtering were performed using xMSanalyzer (v2.0.8) (http://sourceforge.net/projects/xmsanalyzer/) [30]. Each sample was analyzed in triplicate, and coefficient of variation was used to evaluate the quality of all features. Sample quality was evaluated using Pearson correlation within technical replicates. Batch-effect correction was performed using ComBat [31], and xMSannotator v1.3.2 was used to assign computationally derived annotations to metabolic features using the Human Metabolome Database v3.6 [32]. xMSannotator uses a multilevel clustering procedure based on correlation of intensities between metabolic features and grouping based on retention time, isotopes, and adducts. The software assigns annotation scores (0: low confidence to 3: high confidence) to database matches, providing valuable information which can be used to prioritize features for experimental confirmation of molecular identity using tandem mass spectrometry (LC–MS/MS) and authentic standards.

### 2.4. Identification of Discriminatory Features

The purpose of this study was to identify metabolite differences in AMD patients compared to controls, as well as between stages of AMD. Correspondingly, the primary comparisons were between AMD patients and controls, IAMD patients and controls, as well as between NVAMD and IAMD patients. In addition, we performed a reanalysis of the NVAMD patients and controls comparison from our previous study [14] (Figure S1; Table S1). This was performed because the current study used new software versions for data extraction and processing and the analytical approach here differed from our previous study. Therefore, the mass spectral data from samples used in our previous study were re-extracted and processed using updated versions of software. The reanalysis allows for a direct evaluation of metabolite differences across all pairwise comparisons.

A log_2_ transformation was applied to reduce heteroscedasticity and normalize the data. Quantile normalization was performed to minimize between-sample variability [33]. To increase confidence for selection of discriminating metabolites, data were filtered to include only those features present in at least 50% of samples and present in at least 80% of each comparison group. Additionally, features were filtered based on relative standard deviation, removing those with a constant signal across all samples. Following data preprocessing and filtering, partial least-squares discriminant analysis (PLS-DA) was performed with the R package mixOmics [34] to identify discriminating metabolites. Multiple linear regression analysis was performed in parallel, to determine whether age, sex, and AMD status independently influence metabolite intensity. Multiple hypothesis correction for the multiple linear regression analysis was implemented using Benjamini–Hochberg false discovery rate (FDR). Features with a PLS-DA variable importance for projection (VIP) measure ≥ 2 and associated with AMD status at an FDR < 0.1 (*p* < 0.05) from multiple linear regression analyses were included in the final set of discriminatory features. To test the classification performance of the discriminatory features identified for each comparison, 10-fold cross-validation was performed using the *sym* function in R package e1071. The performance was evaluated using the balanced accuracy rate, which is defined as the average of the classification accuracy for each class [14]. Box plots were used to visualize individual discriminatory features in NVAMD and IAMD patients, and non-AMD controls. A Kruskal–Wallis (KW) test was used to determine differences in metabolite intensity between groups.

### 2.5. Metabolite Identification

Discriminatory metabolic features with medium to high confidence matches to known metabolites from xMSannoatator were compared with an in-house library of metabolic features whose molecular identities had previously been confirmed. Using a combination of factors including VIP, fold-change, and biological interest, 15 previously unconfirmed metabolites were selected for further evaluation by LC–MS/MS with the aim of confirming molecular identity. Samples were analyzed using a Thermo LTQ Velos Orbitrap high-resolution mass spectrometer (Thermo Fisher Scientific, San Diego, CA, USA) operated in positive ion mode with 10 min C18 reversed-phase column chromatography and standard source conditions used for the untargeted metabolic profiling. Prior to analysis, plasma proteins were precipitated using acetonitrile and water (2:1 *v*/*v*) and allowed to sit on ice for 30 min. The supernatant was then carefully pipetted for LC-MS/MS analysis. Collision-induced dissociation was accomplished using high purity N_2_ at a normalized collision energy of 35%. Raw LC-MS/MS data were converted to mzXML format using msconvert, and the data were processed using the xcmsSet and xcmsFragments functions in XCMS to extract the LC–MS/MS fragments associated with each parent mass [35,36,37]. The experimental spectra were then compared with the spectra available from mzCloud (https://www.mzcloud.org/) or the in silico predicted spectra using MetFrag [38]. Metabolite identification levels were assigned based on the Metabolomics Standards Initiative (MSI) criteria: (a) confirmation using LC-MS/MS and co-elution with authentic standards (level 1); (b) confirmation by comparing experimental LC-MS/MS spectra with the spectra in mzCloud or in silico predicted spectra retrieved from MetFrag (level 2); (c) annotation at the level of metabolite class (level 3); (d) annotation scores of 2 (medium confidence) or higher from xMSannotator (level 4); (e) accurate mass match or no match (level 5) [39].

### 2.6. Pathway Analysis

To provide further biological context for discriminating metabolites, pathway analyses were performed via Mummichog 2.0 (http://mummichog.org/), a program that combines metabolite annotation and metabolic pathway/network analysis [40,41]. These analyses were performed separately for each comparison using the metabolic features identified by PLS-DA with VIP ≥ 1.5. Using this less stringent VIP threshold allows for greater inclusion of features contributing to the enrichment of pathway differences between groups and prevents information loss [42]. Pathways with a permuted *p* < 0.05 and a minimum feature overlap of three were used for downstream evaluation and analysis.

## 3. Results

### 3.1. Study Population Characteristics

The study population consisted of 191 AMD patients (91 IAMD and 100 NVAMD) and 195 non-AMD controls. Pairwise comparisons of demographic variables, environmental exposures, and comorbidities were performed to determine if any of these differed between groups (Table 1). Age was significantly different in all comparisons, with all AMD patient groups older than controls, and NVAMD patients older than IAMD patients. In this population, there were no significant associations between AMD, NVAMD, or IAMD and sex, BMI, smoking status, diabetes, hypertension, or hyperlipidemia.

### 3.2. High-Resolution Untargeted Metabolomics

Mass spectral data produced by untargeted LC-MS yielded 19,649 ions defined by *m/z* and retention time. Each captured ion had an associated measure of intensity. To increase confidence in selecting the most robust discriminatory metabolites, only those features that met the filtering criteria based on missing values and relative standard deviation were used for downstream analyses in each comparison.

For each comparison described below, PLS-DA and multiple linear regression analyses were performed. Discriminatory metabolic features were those with both a PLS-DA VIP ≥ 2.0 and a linear regression FDR < 0.1 (*p* < 0.05) for association between AMD status and metabolite intensity. For each comparison, 10-fold cross-validation was performed to test the ability of the discriminatory metabolic features to distinguish between the groups.

### 3.3. AMD vs. Controls

**Figure 1:**
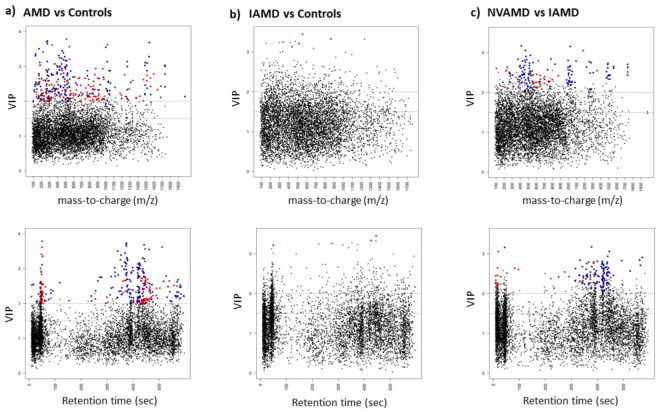
Discriminatory features identified by PLS-DA and linear regression analyses. Manhattan plots displaying significant discriminatory features (PLS-DA VIP ≥ 2 and linear regression FDR < 0.1 (*p* < 0.05)) that were higher (blue dots) or lower (red dots) in the plasma of (**a**) AMD patients compared to controls, (**b**) IAMD patients compared to controls, and (**c**) NVAMD patients compared to IAMD patients. Black dots indicate features that did not meet significance criteria. There are two plots for each comparison—VIP by *m/z* and VIP by Retention time (sec). AMD: age-related macular degeneration; IAMD: intermediate AMD; NVAMD: neovascular AMD; PLS-DA: partial least-squares discriminant analysis; VIP: variable importance for projection.

After data preprocessing and filtering, there were 6419 features available for analysis comparing AMD patients to controls. There were 353 features with a PLS-DA VIP ≥ 2.0. In the linear regression analyses, AMD status was associated with 1135 metabolic features at *p* < 0.05, and 500 met the FDR-corrected significance threshold of < 0.1. A total of 323 metabolic features met significance criteria of a PLS-DA VIP ≥ 2.0 and an FDR < 0.1 (*p* < 0.05) from the linear regression analysis (Table S1). Among these discriminatory metabolic features, 180 were higher, and 143 were lower in AMD patients compared to controls (Figure 1a; Table S1). The 10-fold cross-validation balanced accuracy rate using these 323 discriminatory features was 78.2%.

### 3.4. IAMD Patients vs. Controls

After data preprocessing and filtering, there were 6,337 features available for the IAMD patients versus controls comparison. There were 386 features with a PLS-DA VIP ≥ 2.0. In the linear regression analyses, AMD status was associated with 649 features at *p* < 0.05, though only three met the FDR-corrected significance threshold of < 0.1. These three metabolic features met the significance criteria of a PLS-DA VIP ≥ 2.0 and an FDR < 0.1 (*p* < 0.05) from the linear regression analysis and were all lower in IAMD patients compared to controls (Figure 1b; Table S1). The 10-fold cross-validation balanced accuracy rate using these three features was 54.8%.

### 3.5. NVAMD Patients vs. IAMD Patients

After data preprocessing and filtering, there were 6615 features available for analysis comparing NVAMD patients to IAMD patients. There were 328 features with a PLS-DA VIP ≥ 2.0. In the linear regression analyses, AMD status was associated with 1,024 metabolic features at *p* < 0.05, and 172 met the FDR-corrected significance threshold of < 0.1. A total of 158 metabolic features met the significance criteria of a PLS-DA VIP ≥ 2.0 and an FDR < 0.1 (*p* < 0.05) from the linear regression analysis (Table S1). Among these discriminatory features, levels of 120 were higher and levels of 38 were lower in NVAMD patients compared to IAMD patients (Figure 1c; Table S1). The 10-fold cross-validation balanced accuracy rate using these 158 discriminatory features was 77.8%.

### 3.6. Metabolite Identification

Among the three comparisons, there were 435 unique significant discriminatory features identified (Table S2). To determine molecular identities, discriminatory features were compared with an in-house library of metabolic features whose molecular identities were previously verified. In addition, a subset of discriminatory features whose molecular identities could not be confirmed in the database comparison was evaluated by LC–MS/MS analysis. The molecular identity of 14 discriminatory features was confirmed at MSI metabolite identification level 1 or 2 (Table 2). Of these discriminatory features, the molecular identities of 11 had been previously verified [14,23,32,43,44], while three were newly confirmed in this study. The 14 metabolites whose molecular identities were confirmed could be broadly categorized into four classes: acylcarnitines, lipids and related metabolites, amino acid-related metabolites, and steroids and steroid derivatives.

**Figure 2:**
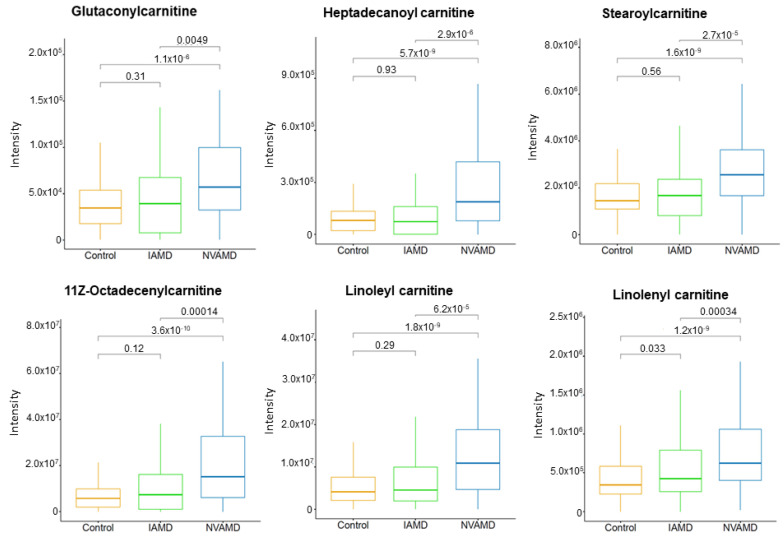
Medium- and long-chain plasma acylcarnitines altered in AMD patients. Plasma levels of six acylcarnitines that were higher in NVAMD patients compared to either IAMD patients or controls. Kruskal–Wallis tests were used for pairwise comparisons and *p*-values are shown above comparison brackets. The molecular identities of these acylcarnitines have been confirmed at MSI level 2.

Six of the LC–MS/MS-confirmed metabolites were acylcarnitines (Table 2). Of these, two were approximately 1.5-fold higher in AMD patients compared to controls and four were between 1.7- and 2.0-fold higher in NVAMD patients compared to IAMD patients. Five of the acylcarnitines were medium- or long-chain acylcarnitine species (Figure 2). In addition, there were seven discriminatory features with medium or high confidence matches to acylcarnitines that similarly displayed higher levels with more advanced disease (Figure S2).

**Figure 3:**
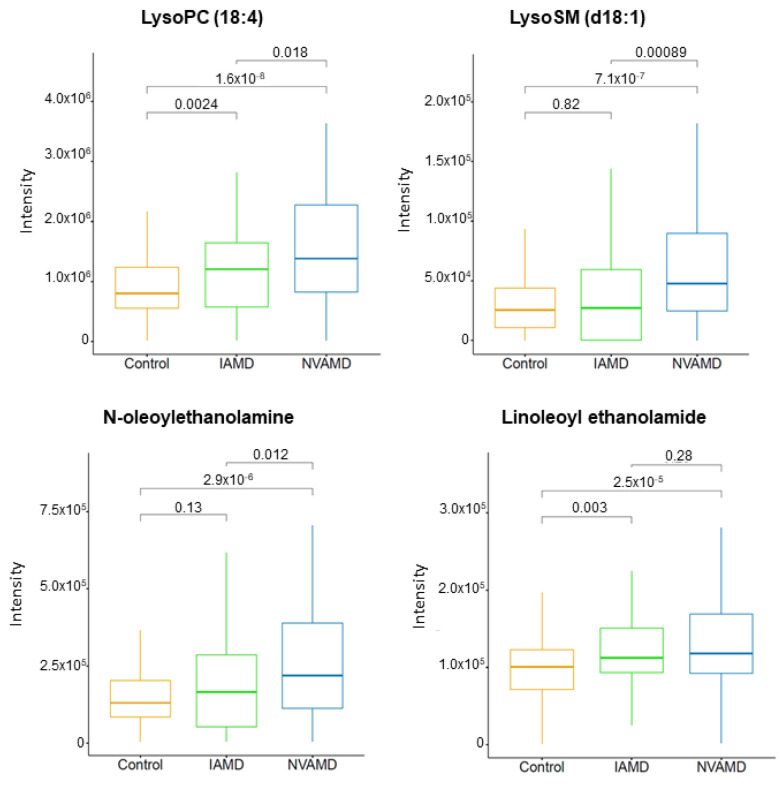
Plasma lipid metabolites altered in AMD patients. Plasma levels of LysoPC (18:4) and LysoSM (d18:1) were higher in NVAMD patients compared with IAMD patients or controls Plasma levels of N-oleoylethanolamine and linoleoyl ethanolamide were higher in NVAMD patients compared to controls. Kruskal–Wallis tests were used for pairwise comparisons and *p*-values are shown above comparison brackets. The molecular identities of these metabolites have been confirmed at MSI level 1 or 2.

There were four LC–MS/MS-confirmed lipids and related metabolites among the discriminatory features in the AMD patient groups (Table 2). Lysosphingomyelin (d18:1), an intermediate in sphingolipid metabolism, was more than 2.0-fold higher in NVAMD patients compared to IAMD patients, and lysophosphatidylcholine (18:4) was approximately 1.5-fold higher in AMD patients compared to controls. There were also two n-acylethanolamines that were higher in AMD patients compared to controls (Table 2; Figure 3).

**Figure 4:**
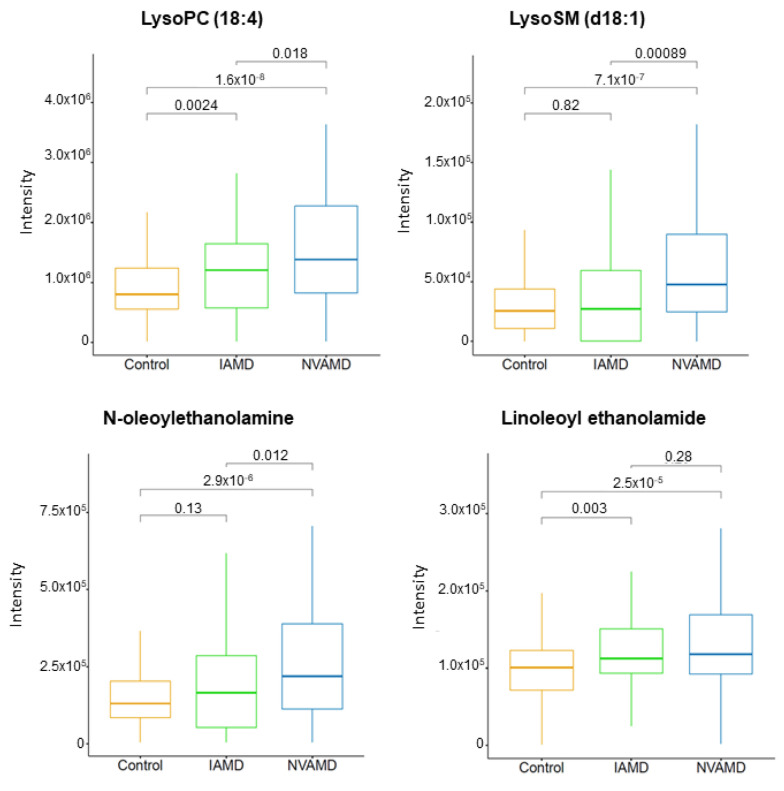
Plasma steroid-related metabolites altered in AMD patients. Plasma levels of the steroid-related metabolites cortexolone and 25-hydroxyvitamin D2 were altered in AMD patients. Kruskal–Wallis tests were used for pairwise comparisons and *p*-values are shown above comparison brackets. The molecular identities of these metabolites have been confirmed at MSI level 2.

Finally, there were two steroid-related metabolites identified among the discriminatory metabolites (Table 2). Cortexolone was approximately 3.0-fold higher in AMD patients compared with controls, and 25-hydroxyvitamin D2 was approximately 2.0-fold higher in NVAMD patients compared with IAMD patients (Table 2; Figure 4).

### 3.7. Pathway Analysis

**Figure 5:**
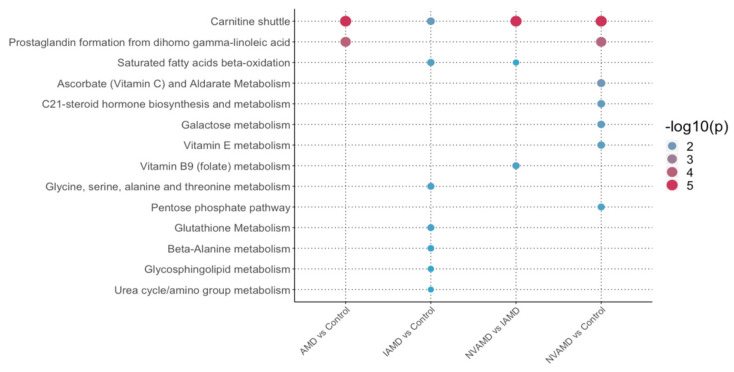
Pathways altered in AMD patients. Summary of the pathways altered in AMD patients compared to controls, as well as across stages of AMD. Pathway analysis was performed using Mummichog 2.0 on discriminatory metabolic features identified by PLS-DA at VIP ≥ 1.5. The NVAMD versus Control results are an updated reanalysis of data from Mitchell SL et al. 2018 and are included here for comparison. Size and color of circles indicate significance level. AMD: age-related macular degeneration; IAMD: intermediate AMD; NVAMD: neovascular AMD.

Pathway analysis was performed for each comparison using metabolic features identified by PLS-DA with a VIP ≥ 1.5. Applying a less stringent VIP threshold in pathway analysis allows for broader inclusion of features to better identify pathways that are altered in the patient groups. Multiple lipid metabolism pathways were identified in the pathway analysis. The *carnitine shuttle* pathway was significant in all comparisons (Figure 5), and the *prostaglandin formation from dihomo-gamma-linoleic acid* was altered in AMD patients compared to controls. The *saturated fatty acids beta-oxidation* pathway was significant in the IAMD versus controls and the NVAMD versus IAMD comparisons, and the *glycosphingolipid metabolism* pathway was altered in IAMD patients compared with controls (Figure 5). In addition, there were four amino acid metabolism pathways altered in IAMD patients compared with controls, and the *vitamin B9 (folate) metabolism* pathway was altered in NVAMD patients compared with IAMD patients (Figure 5).

## 4. Discussion

Presently, the mechanisms underlying AMD development and progression remain largely undefined. Despite advances in identifying AMD risk factors, precisely distinguishing which patients will progress to advanced disease remains a difficult challenge. Determining the etiologic factors affecting development of NVAMD could advance novel treatments and potentially prevent disease progression. With the goal of uncovering valuable insight into disease pathophysiology, we applied untargeted, high-resolution metabolomics to identify metabolites and metabolic pathways altered in IAMD and NVAMD patients.

Among the comparisons, we identified more than 400 unique discriminatory metabolites, none of which was common to all comparisons. In the IAMD versus controls comparison, only three discriminatory features were identified. This represents fewer than 0.1% of the features analyzed in this comparison. In the NVAMD versus IAMD and AMD versus controls comparisons, 2.4% and 5.0% of analyzed features, respectively, were identified as discriminatory. In the NVAMD versus controls reanalysis, 5.0% of analyzed features were identified as discriminatory. Among the four comparisons, similar numbers of features were available for analysis. In addition, the majority (63%) of the discriminatory features identified in the AMD versus controls comparison were also discriminatory features in the NVAMD versus controls reanalysis. Taken together, these data suggest that there are minimal plasma metabolic differences between IAMD patients and controls, and many of the differences observed between AMD patients and controls are largely due to alterations in NVAMD patients.

We observed higher plasma levels of multiple acylcarnitines in NVAMD patients compared with IAMD patients. Box plots of several of the individual acylcarnitines illustrate a progressive increase from non-AMD to IAMD to NVAMD. In a prior study, we determined that the *carnitine shuttle* pathway was altered in NVMAD patients compared to controls [14], and the updated analysis of these data here also identified this pathway. Other metabolomics studies have reported altered plasma acylcarnitines in AMD patients [12,13,15], providing further support that this pathway may be important in AMD. In the current study, pathway analysis results demonstrate that the *carnitine shuttle* pathway is also significantly altered in NVAMD patients compared to IAMD patients, which has not been previously reported. The carnitine shuttle is essential for mitochondrial β-oxidation of fatty acids, and these alterations indicate that progressive dysfunction of mitochondrial fatty acid metabolism could be a key contributing factor to AMD progression. Indeed, there are links between fatty acid oxidation and regulation of angiogenesis. Kalucka et al. demonstrated that fatty acid oxidation is critical for maintaining redox homeostasis and preventing dysfunction in quiescent endothelial cells [45]. Inhibition of fatty acid oxidation via the rate-limiting enzyme carnitine palmitoyltransferase 1 (CPT1) repressed proliferation and neovascularization in endothelial cells and inhibited pathological ocular angiogenesis in mice [46]. Combined with prior studies, our results suggest that the *carnitine shuttle* pathway is altered to a greater degree in NVAMD patients and point to the need for longitudinal studies for verification, as well as studies to test for potential benefits of mitochondria-related interventions.

Multiple studies have reported changes in plasma phospholipids in AMD patients, including a recent investigation which detected differences in the glycerophospholipid metabolism pathway in AMD patients compared with controls [13,15,16]. In the current study, we identified three phospholipid-related metabolites that were higher in AMD patients compared with controls. Two of these were n-acylethanolamines, which are components of n-acylphosphatidylethanolamine, a membrane-bound phospholipid. N-acylethanolamines may accumulate under certain conditions including glutamate excitotoxicity, ischemia, and inflammation [47,48,49,50,51], all of which may contribute to the pathophysiology of AMD. Studies have demonstrated that certain n-acylethanolamines are neuroprotective [49,50]. For example, N-linoleoyl ethanolamine treatment conferred protection to retinal ganglion cells in mouse retinal explants treated to induce glutamate excitotoxicity [49]. N-acylethanolamines and their receptors are found in the retina, and thus represent a potential therapeutic mechanism for neuroprotection in ocular diseases [47,49,51].

Overall, pathway analysis revealed nine metabolic pathways altered in patients with NVAMD, IAMD, or AMD in general. Most of the pathways could be categorized into three groups: lipid metabolism, amino acid metabolism, and vitamin metabolism. These results are broadly consistent with previous studies reporting alterations in lipids, amino acids, and vitamins in the plasma of AMD patients [10,11,12,13,14,15,16,17,52]. Although only three individual metabolic features were identified as discriminatory in the IAMD versus controls comparison, pathway analysis, which included features based on less stringent criteria, revealed multiple *amino acid metabolism* pathways that were specifically altered in IAMD patients compared with controls, suggesting that dysregulation of amino acid metabolism may occur in earlier stages of AMD. A recent NMR-based, targeted AMD metabolomics study reported changes in the levels of multiple amino acids including alanine [17], which we identified via pathway analysis as altered in IAMD patients. As with the alterations in acylcarnitines, these differences in amino acid metabolism could also reflect changes in mitochondrial activities. For instance, changes in amino acid metabolism are linked to mitochondrial function and energy balance through glutamate, aspartate, glycine, serine and alanine metabolism, as well as through the urea cycle. Threonine and the β-alanine pathways also provide links to mitochondrial fatty acid metabolism. Thus, changes in *amino acid metabolism* pathways in IAMD could be important in disease development.

A major strength of this study is the well-defined cohort of nearly 400 AMD patients and controls that includes both IAMD and NVAMD patients, allowing for comparisons between different stages of disease. Another strength is the use of untargeted, high-resolution LC–MS to provide broad coverage of low- and high-abundance metabolites. Metabolite identification is a major bottleneck in untargeted metabolomics studies, due in part to low abundance of metabolites of interest and multiple isomeric matches or no matches in metabolite databases [53,54,55]. Given this, we employed a combination of computational methods to enhance confidence in metabolite annotations and assign MSI metabolite identification levels. We identified 11 metabolites whose molecular identity had been previously verified, and we confirmed the identity of an additional three metabolites via targeted LC–MS/MS. The molecular identities of these 14 metabolites were all confirmed at MSI identification level of 1 or 2. Additional targeted studies are needed to confirm molecular identities of discriminatory metabolites with a current MSI identification level of 3 or higher.

In conclusion, this study demonstrates that the plasma metabolic profiles of IAMD patients are more similar to controls than to NVAMD patients. These results suggest that when comparing a group of AMD patients that includes IAMD and NVAMD patients to non-AMD controls, the metabolomic differences identified may be largely a consequence of changes in the NVAMD patients. Further studies are necessary to determine how the plasma metabolite differences between stages of AMD correlate to the eye and contribute to the pathophysiology of this disease. Longitudinal studies are an important direction for future investigations, as having data from multiple time points can provide deeper mechanistic insight into disease progression.

## Figures and Tables

**Table 1:** Study Population Characteristics.

Characteristic	Controls (n = 195)	AMD (n = 191)	IAMD (n = 91)	NVAMD (n = 100)
Age, years	71.9 ± 6.5	77.6 ± 7.3 *	75.7 ± 7.5 *	79.2 ± 6.8 *^,†^
Female, %	63.6	64.4	63.0	65.0
Smokers, %	50.3	57.1	52.8	61.0
BMI, kg/m^2^	26.9 ± 5.0	26.5 ± 4.5	26.1 ± 4.8	26.8 ± 4.2
Diabetes, %	17.7	20.8	17.8	23.3
Hypertension, %	51.1	54.0	46.0	60.9
Hyperlipidemia, %	53.9	45.9	41.7	49.4

Age and BMI are presented as the mean ± standard deviation. Welch’s t-test was used to test for differences in age and BMI between groups: AMD patients versus controls, IAMD patients versus controls, NVAMD patients versus IAMD patients, and NVAMD patients versus controls. For all other characteristics, a chi-squared test was used to test for differences between groups. BMI: body mass index; AMD: age-related macular degeneration; IAMD: intermediate AMD; NVAMD: neovascular AMD. * Significantly different compared to controls (*p* < 0.05). ^†^ Significantly different compared to IAMD patients (*p* < 0.05).

**Table 2:** Discriminatory metabolic features with confirmed molecular identities.

*m*/*z*	RT (sec)	Verified Metabolite or Metabolite Class	Metabolite Identification Level	AMD/ Controls Fold Change	IAMD/ Controls Fold Change	NVAMD/ IAMD Fold Change
***Acylcarnitines***						
414.3568	377.7	Heptadecanoyl carnitine	2	-	-	2.08
426.3568	366.5	11Z-Octadecenylcarnitine	2	-	-	1.74
424.3419	345.9	Linoleyl carnitine	2	-	-	1.75
422.3270	330.5	Linolenyl carnitine *	2	1.63	-	-
274.1263	373.8	Glutaconylcarnitine	2	1.49	-	-
428.3721	392.6	Stearoylcarnitine	2	-	-	1.83
***Lipid metabolites***						
466.3518	332.9	LysoSM (d18:1) *	2	-	-	2.34
516.3057	376.2	LysoPC (18:4) *	2	1.47	-	-
324.2893	471.0	Linoleoyl ethanolamide	2	1.32	-	-
326.3045	526.9	N-Oleoylethanolamine	2	1.37	-	-
***Steroids and steroid derivatives***						
413.3451	351.0	25-Hydroxyvitamin D2	2	-	-	2.16
347.2213	271.9	Cortexolone	2	3.12	-	-
***Amino acid-related metabolites***						
130.0499	51.9	Pyroglutamic acid	1	1.52	-	-
209.0910	234.1	Kynurenine	1	-	-	0.72

Discriminatory metabolic features were identified by PLS-DA (VIP ≥ 2.0) and linear regression analysis (FDR < 0.1 and *p* < 0.05). To verify their molecular identities, features were compared with an in-house library of metabolic features whose molecular identities had previously been confirmed. A subset of previously unconfirmed metabolites was evaluated by LC–MS/MS. * molecular identity newly confirmed in this study; *m/z*: mass-to-charge ratio; RT (sec): retention time (seconds).

## Data Availability

The data presented in this study are available in the article and supplementary material.

## Supplementary Materials

The following are available online at https://www.mdpi.com/article/10.3390/cells10113141/s1, Figure S1: Features that discriminate between NVAMD patients and controls, Figure S2: Putative acylcarnitines altered in AMD patients, Table S1: Discriminatory features identified for each comparison, Table S2: Overlap of discriminatory features for all comparisons.

## Abbreviations

AMD: age-related macular degeneration; IAMD: intermediate age-related macular degeneration; NVAMD: neovascular age-related macular degeneration; BMI: body mass index; LC–MS: liquid chromatography–mass spectrometry; CARMS: Clinical Age-Related Maculopathy Scale; *m/z*: mass-to-charge ratio; RT: retention time; LC–MS/MS: tandem mass spectrometry; PLS-DA: partial least-squares discriminant analysis; VIP: variable importance for projection; MSI: metabolomics standards initiative.
