# Supplementary material for: Plasma Metabolomics of Intermediate and Neovascular Age-Related Macular Degeneration Patients
Source: Cells. 2021 Nov 12;10(11):3141. doi: 10.3390/cells10113141 (PMC8624113; doi:10.3390/cells10113141)

Supplementary material

NVAMD patients vs controls comparison

We performed PLS-DA and linear regression on the 7,012 features available for analysis comparing NVAMD patients to controls. As outlined in the Methods, these results are a reanalysis of data from our previous study [13] using updated software versions for data extraction and processing. The reanalysis allows for direct evaluation of metabolic differences across all pairwise comparisons. There were 360 features with a VIP ≥ 2.0. In the linear regression analyses, 1,690 metabolic features were associated with AMD status at $p < 0.05$ and 1,070 met the FDR-corrected significance threshold of < 0.1. A total of 355 metabolic features met significance criteria of a PLS-DA VIP ≥ 2.0 and an FDR < 0.1 ($p < 0.05$) from the linear regression analysis (**Table S1**). Of the discriminatory metabolic features, 231 were higher and 124 were lower in NVAMD patients compared to controls (**Figure S1; Table S1**). The 10-fold cross validation balanced accuracy rate using these 355 discriminatory features was 70.7%.

Pathway analysis identified seven pathways altered in NVAMD patients compared to controls (**Figure 5**). The most significant were the *carnitine shuttle* and *prostaglandin formation from dihomo gamma-linoleic acid* pathways. The *carnitine shuttle* pathway was identified in the previous analysis of these data (Mitchell SL et al 2018). Additional pathways included *pentose phosphate* and *galactose metabolism*, as well as multiple vitamin metabolism pathways.

Figure S1: Features that discriminate between NVAMD patients and controls

Manhattan plots displaying significant discriminatory features (PLS-DA VIP ≥ 2 & linear regression FDR < 0.1 ($p < 0.05$)) that were higher (blue dots) or lower (red dots) in the plasma of NVAMD patients compared to controls. Black dots indicate features that did not meet significance criteria. Plots with VIP by *m/z* and VIP by Retention time (sec). NVAMD: neovascular AMD; PLS-DA: partial least squares discriminant analysis; VIP: variable importance for projection; *m/z:* mass-to-charge ratio

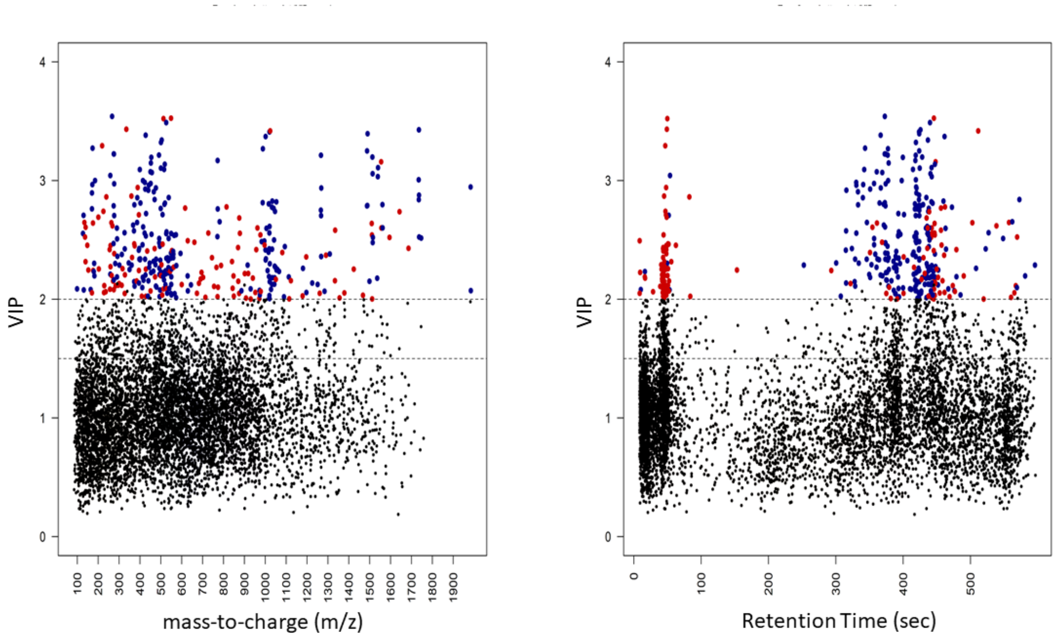

Figure S2: Putative acylcarnitines altered in AMD patients

Plasma levels of discriminatory features that had medium or high confidence matches to acylcarnitines are altered in AMD patients. Kruskal-Wallis tests were used for pairwise comparison of plasma levels of these metabolites and p-values are shown above comparison brackets. Discriminatory features are labeled with mass-to-charge ratio and retention time (m/z_RT) and have an MSI identification level of 3 or 4.

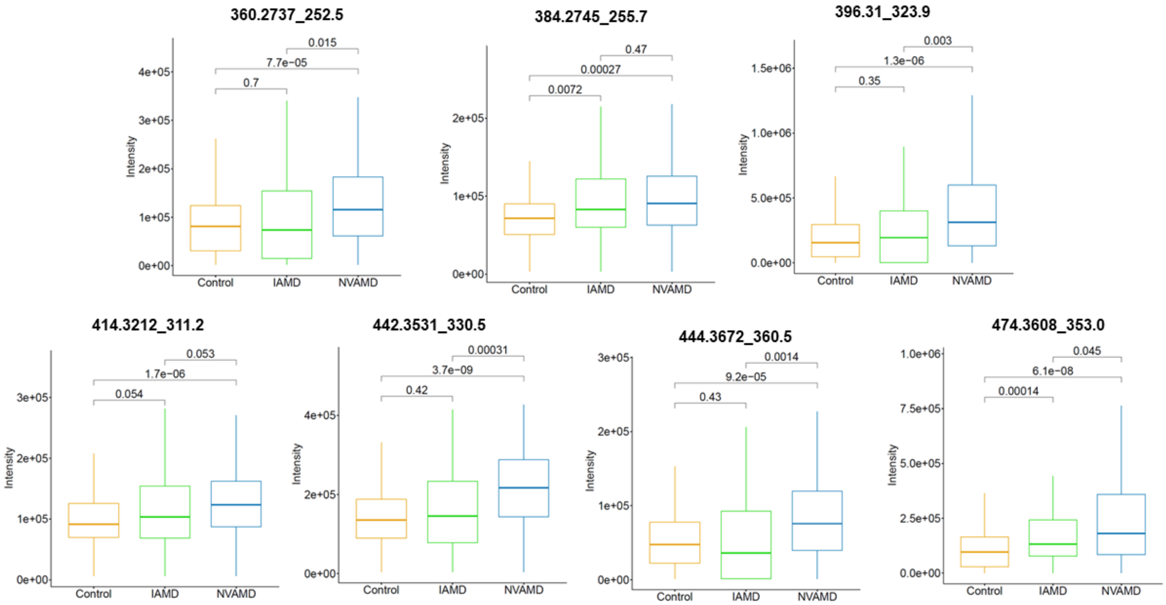

Supplement: Supplementary file 1 [file cells-10-03141-s001.zip › Mitchell_etal_Supplementary_Figures_10Nov2021.pdf]
